# The Consequence of Complete Dentures on Quality of Life of Edentulous Patients in the South-Indian Population Based on Educational and Socioeconomic Grades

**DOI:** 10.7759/cureus.6923

**Published:** 2020-02-09

**Authors:** Madhan Seenivasan, Fathima Banu, Athiban Inbarajan, Parthasarathy Natarajan, Shanmuganathan Natarajan, Anand Kumar V, Karthigeyan J

**Affiliations:** 1 Prosthodontics, Sri Ramachandra University, Chennai, IND; 2 Prosthodontics, Faculty of Dental Sciences, Sri Ramachandra Institute of Higher Education and Research, Chennai, IND

**Keywords:** complete denture satisfaction, socioeconomic status, education level, psychology, edentulism, quality of life, complete denture

## Abstract

Purpose

The purpose of this study was to establish the level of denture satisfaction with socio-demographic variables and educational status of the patients rehabilitated with complete denture.

Materials and method

A total number of 250 completely edentulous patients were selected who fulfilled the inclusion and exclusion criteria. The patients had no past medical history which affects the oral condition; they were first-time denture wearers with period of edentulousness altering between six months to one year and were in the age group of 40-50 years, and were willingly involved in the study. The subjects were grouped according to their socioeconomic status such as employment, education and income level. The correlations were statistically determined using regression analysis.

Results

Statistical analysis was done using Statistical Package for Social Sciences (SPSS, Chicago, Illinois, USA), version 16.0. The significance of percentage error of the two groups was tested by Student t test and p value denoted level of significance (p<.05). Based on the education level, 30.47% of the population were under primary level of education, 57.82% completed higher secondary education and 11.72% of the population were graduates. Based on employment status, 53.12% of the population was unemployed, 32.03% were employed while 14.84% of the population were pensioners. Based on income per month, the population was classified as 6.25%, 31.25%, 21.09%, 22.66%, 18.75% for no income, less than 3000, 5000, 8000 and more than 10000 respectively. Psychological comfort, social ability, and functional improvement was better with higher secondary education level, employed and lower income individuals.

Conclusion

Rehabilitation of an elderly individual not only includes clinician skills but also the personal perception by the patient. The study concludes that the though there was no statistically significant difference, the individual with secondary level of education and with employed low socioeconomic status had better denture satisfaction than the other category.

## Introduction

Management of edentulous patients during rehabilitation with complete dentures is still lacking with respect to patients based on educational level and socioeconomic status [1]. The need for thought of oral health-related quality of life (QoL) has been increasingly accepted over the last decades, and many studies highlight the psychosocial impacts of oral conditions. This study is based on considerations in making of complete dentures of different socio-demographic variables such as age, gender, literacy level, socio-economic and educational status may affect satisfaction towards dentures. To assess this, a consistent questionnaire that included questions from domains such as mastication, appearance, speech, comfort, health, denture care and social status is used to establish level of denture satisfaction with socio-demographic variables and educational status of the patients.

The removable denture prosthesis (RDP) must be able to restore the chewing function, aesthetics and phonetics to compensate partial edentulism [2]. Considering the biomechanics involved allows the specialist to design a removable partial denture prosthesis by establishing and maintaining lift, stabilization and retention termed the Housset triad. With these imperatives taken into account, and depending on the number of teeth lost and the type of edentulous areas bounded by remaining teeth or without posterior tooth support, the constraints on the prosthesis will be different and functional rehabilitation altered. One such method is by measuring food bolus granulometry before swallowing, associated with analysis of the kinematic parameters developed to distinguish patients with normal mastication from those with badly impaired mastication [3]. Impaired chewing function leads to raise of food bolus particle size, measured by the median particle size of the food bolus at swallowing. It has been revealed that adults with impaired mastication could be distinguished from those with normal function if the median particle size of the bolus that they produced, when chewing raw carrot reached a cut-off value of 4 mm, called the masticatory normative indicator (MNI) [4]. The adjustment of chewing behaviour to food hardness can also characterize healthy mastication. Adaptation to increasing food hardness marks in an augmented number of chewing cycles and an increase in the chewing sequence duration, with no modification of the chewing frequency (number of cycles per second) in healthy subjects [5,6]. The mean chewing frequency is slowed down in subjects with chewing deficiencies while eating any type of resistant food. Earlier studies on the chewing ability of dentally impaired subjects showed that a decrease in the number of functionally paired teeth and oral rehabilitation with removable dentures were linked to a decreased masticatory values [7,8]. But, the physiological impact of RDP rehabilitation has been seldom studied. Also, the objective of this work was to estimate the impact of partial edentulous areas rehabilitation by removable partial denture prosthesis with a socioeconomic and educational point of view.

Beside the therapist's ability and the quality of dentures, individual factors connected with the patient are very important for the final satisfaction with dentures. Patients are sometimes not satisfied with the constructions which are best, according to the therapist's judgment. Satisfaction with dentures seems to have multiclausal character. According to the results of Frank's studies, the most frequent areas of dissatisfaction were as follows: fit (33.6%), mastication (29.5%), natural tooth problems (26.3%), overall perception (26,2%), oral cleanliness (20.4%), speech (17.9%), appearance (17.8%), denture cleanliness (15.3%) and odour (13.2%) [9,10]. In different studies concerning satisfaction or dissatisfaction with partial removable dentures, more concern was placed on upper partial dentures. Dentists consider dentures to be successful when they meet certain methodological standards, whereas patients assess them from the viewpoint of personal satisfaction. The capability to adapt to new dentures will usually reduce in proportion to the individual status. To assess this, a consistent questionnaire that included questions from domains such as mastication, appearance, speech, comfort, health, denture care and social status was used to determine level of denture satisfaction with socio-demographic variables of completely edentulous patients rehabilitated with prosthesis.

## Materials and methods

The study was conducted at the Department of Prosthodontics, Sri Ramachandra Institute of Higher Education and Research (SRIHER) with the approval of the ethics committee. A total number of 250 completely edentulous patients were selected who fulfilled the inclusion and exclusion criteria. The patients had no past medical history which affects the oral condition, first-time denture wearers, period of edentulousness altering between six months to one year and Class I edentulous state as classified by American College of Prosthodontics and in the age group of 40-50 years who were willingly involved in the study was selected. The subjects were grouped according to their socioeconomic status such as employment, education and income level. According to the employment level, they were divided into Employed, Self-Employed, Unemployed and Pensioners. According to education level, they were separated as Primary (till standard five), Secondary (till standard nine), and Tertiary education. Income level they are divided into low, middle, and high-income group. The removable prosthesis was fabricated in the Department of Prosthodontics and their quality were assessed based on the method given by Sato et al [11]. The patients were interviewed at 2-3 months post-treatment. A single person conducted all the questionnaire surveys to reduce the discrepancy. A standardized questionnaire, with 19 questions based on denture satisfaction level and masticatory capacity in the domains of Functional limitation, Psychological discomfort, Psychological disability, and Social disability was administered [12]. All the questions were calculated in scale of satisfied, moderately satisfied and hardly ever. The denture satisfaction questions were only asked at the post-treatment interview and relevant to the satisfaction of their new maxillary/mandibular complete dentures the patients received according to the Likert scale. Statistical analysis was done using Statistical Package for Social Sciences (SPSS, Chicago, Illinois, USA), version 16.0. Significance of percentage error of two groups was tested by Student t test and p value denoted level of significance (p<.05).

## Results

Distribution of sample

Based on the education level, 30.47% of the population were under primary level of education, 57.82% of the population have done higher secondary education and 11.72% of the population were graduates. Based on employment status, 53.12% of population was unemployed, 32.03% of the population were employed while 14.84% of the population were pensioners. Based on income per month population were classified as 6.25%, 31.25%, 21.09%, 22.66%, 18.75% for no income, less than 3000, 5000, 8000 and more than 10000 respectively.

Psychological discomfort

On postoperative evaluation based on education, the satisfactory level for psychological comfort was higher for higher secondary educated persons followed by primary education and graduate persons. The distribution of sample was Higher secondary - 41, primary - 22 and graduate - 8 for satisfaction level questionnaire (SAQ)4 and SAQ5, Higher secondary - 55, primary - 29 and graduate - 10 for SAQ9. Based on masticatory ability, the distribution of sample was Higher secondary - 42, primary - 20 and graduate - 2 for masticatory ability questionnaire (MCQ)9 and Higher secondary - 56, primary - 24 and graduate - 12 for MCQ12. Though there was no statistical significance, the psychological comfort was better with Higher secondary education level (Table 1). 

**Table 1:** Psychological Discomfort Based on Education SAQ - Satisfaction level questionnaire, MCQ - Masticatory ability questionnaire

Questionnaire	Education level	Satisfied	Moderately Satisfied	Not Satisfied	Pearson Chi-Square P value
SAQ4	Primary	22	10	1	0.119659
	Higher Secondary	41	15	17	
	Graduate	8	3	4	
SAQ5	Primary	21	10	2	0.105918
	Higher Secondary	44	12	18	
	Graduate	8	5	2	
SAQ9	Primary	29	2	2	0.066393
	Higher Secondary	55	9	10	
	Graduate	10	0	5	
MCQ9	Primary	20	13	0	0.818271
	Higher Secondary	42	30	2	
	Graduate	8	7	0	
MCQ 12	Primary	24	8	1	0.867339
	Higher Secondary	56	16	2	
	Graduate	12	2	1	

On postoperative evaluation based on employment status, the satisfactory level for psychological comfort was more for employed persons followed by unemployed and pensioner persons. The distribution of sample was Employed - 47, Unemployed - 16 and Pensioner - 10 for SAQ4 and Employed-50, Unemployed - 15 and Pensioner - 12 for SAQ5, Employed - 68, Unemployed - 18 and Pensioner - 13 for SAQ9. Based on masticatory ability, the distribution of sample was Employed - 49, Unemployed - 15 and Pensioner - 9 for MCQ9 and Employed-61, Unemployed - 19 and Pensioner - 16 for MCQ12. Though there was no statistical significance, the psychological comfort was better with employed persons (Table 2). 

**Table 2:** Psychological Discomfort Based on Employment SAQ - Satisfaction level questionnaire, MCQ - Masticatory ability questionnaire

Questionnaire	Employment Status	Satisfied	Moderately Satisfied	Not Satisfied	Pearson Chi-Square P value
SAQ4	Unemployed	16	4	5	0.818145
	Employed	47	21	14	
	Pensioner	10	6	3	
SAQ5	Unemployed	15	6	4	0.518909
	Employed	50	16	17	
	Pensioner	12	6	1	
SAQ9	Unemployed	18	2	5	0.538068
	Employed	68	7	8	
	Pensioner	13	2	4	
MCQ9	Unemployed	15	10	0	0.696123
	Employed	49	32	2	
	Pensioner	9	10	0	
MCQ12	Unemployed	19	6	0	0.605909
	Employed	61	18	4	
	Pensioner	16	3	0	

On postoperative evaluation based on income, the satisfactory stage for psychological comfort were higher for low income individuals followed by upper middle class, lower middle class and higher class individuals. The distribution of sample was lower class -25, upper middle class -17, lower middle class-16 and higher class - 13 for SAQ4 and lower class -25, upper middle class -17, lower middle class-19 and higher class - 12 for SAQ5, lower class -34, upper middle class -25, lower middle class-18 and higher class - 17 for SAQ9. Based on masticatory ability, the distribution of sample was lower class -23, upper middle class -19, lower middle class-14 and higher class - 14 for MCQ9 and lower class -29, upper middle class -20, lower middle class-21 and higher class - 21 for MCQ12. Although there was no statistical significance, the psychological comfort was more with lower income individual, while it was very less with no income particpants (Table 3).

**Table 3:** Psycological Discomfort Based on Income SAQ - Satisfaction level questionnaire, MCQ - Masticatory ability questionnaire

Questionnaire	Income per month	Satisfied	Moderately Satisfied	Not Satisfied	Pearson Chi-Square P value
SAQ4	Nil	0	0	1	0.306507
	3000	25	11	4	
	5000	16	7	4	
	8000	17	7	4	
	10000&above	13	4	7	
SAQ5	Nil	0	0	1	0.342548
	3000	25	9	6	
	5000	17	5	5	
	8000	19	8	2	
	10000&above	12	6	6	
SAQ9	Nil	1	0	0	0.150084
	3000	34	2	4	
	5000	18	6	3	
	8000	25	2	2	
	10000&above	17	1	6	
MCQ9	Nil	0	1	1	0.649235
	3000	23	17	0	
	5000	14	13	2	
	8000	19	10	1	
	10000&above	14	10	1	
MCQ12	Nil	1	0	0	0.847432
	3000	29	10	1	
	5000	21	6	0	
	8000	20	8	1	
	10000&above	21	3	0	

Social disability

On postoperative evaluation based on education, the satisfactory stage for social ability were higher for Higher secondary educated individuals followed by primary education and graduate individuals. The distribution of sample was Higher secondary-49, primary - 25 and graduate - 9 for SAQ3, Higher secondary-51, primary - 23 and graduate - 8 for SAQ7. Based on masticatory ability, the distribution of sample was Higher secondary-49, primary - 24 and graduate - 12 for MCQ10, Higher secondary-57, primary - 23 and graduate - 13 for MCQ11 and Higher secondary-54, primary - 25 and graduate - 9 for MCQ13. Though there was no statistical significance, the social ability was better with Higher secondary education level (Table 4).

**Table 4:** Social Disability Based on Education SAQ - Satisfaction level questionnaire, MCQ - Masticatory ability questionnaire

Questionnaire	Education level	Satisfied	Moderately Satisfied	Not Satisfied	Pearson Chi-Square P value
SAQ3	Primary	25	6	2	0.564003
	Higher Secondary	49	12	13	
	Graduate	9	3	3	
SAQ7	Primary	23	7	3	0.295081
	Higher Secondary	51	12	11	
	Graduate	8	2	5	
MCQ10	Primary	24	8	1	0.725246
	Higher Secondary	49	24	1	
	Graduate	12	3	0	
MCQ11	Primary	23	9	1	0.532581
	Higher Secondary	57	15	2	
	Graduate	13	1	1	
MCQ13	Primary	25	6	1	0.357926
	Higher Secondary	54	18	2	
	Graduate	9	4	2	

On postoperative assessment based on employment status, the satisfactory level for social ability were higher for employed individuals followed by unemployed and pensioner individuals. The distribution of sample was Employed-57, Unemployed - 16 and Pensioner - 13 for SAQ3 and Employed-57, Unemployed - 17 and Pensioner - 12 for SAQ7. Based on masticatory ability, the distribution of sample was Employed-58, Unemployed - 16 and Pensioner -15 for MCQ10, Employed-61, Unemployed - 19 and Pensioner - 17 for MCQ11 and Employed-61, Unemployed - 17 and Pensioner - 15 for MCQ13. Though there was no statistical significance, the social ability was better with employed individuals (Table 5).

**Table 5:** Social Disability Based on Employment SAQ - Satisfaction level questionnaire, MCQ - Masticatory ability questionnaire

Questionnaire	Employment Status	Satisfied	Moderately Satisfied	Not Satisfied	Pearson Chi-Square P value
SAQ3	Unemployed	16	5	4	0.94543
	Employed	57	13	13	
	Pensioner	13	4	2	
SAQ7	Unemployed	17	3	5	0.869279
	Employed	57	15	11	
	Pensioner	12	4	3	
MCQ10	Unemployed	16	9	0	0.682443
	Employed	58	23	2	
	Pensioner	15	4	0	
MCQ11	Unemployed	19	6	0	0.438342
	Employed	61	18	4	
	Pensioner	17	2	0	
MCQ13	Unemployed	17	8	0	0.567791
	Employed	61	17	4	
	Pensioner	15	3	1	

On postoperative evaluation based on income, the satisfactory level for social ability were higher for low income individuals followed by upper middle class, lower middle class and higher class individuals. The distribution of sample was lower class -27, upper middle class -24, lower middle class-19 and higher class - 13 for SAQ3 and lower class -26, upper middle class -24, lower middle class-19 and higher class - 13 for SAQ7. Based on masticatory ability, the distribution of sample was lower class -28, upper middle class -20, lower middle class-19 and higher class - 18 for MCQ10, lower class -28, upper middle class -23, lower middle class-20 and higher class - 22 for MCQ11 and lower class -32, upper middle class -24, lower middle class-20 and higher class - 14 for MCQ13. Though there was no statistical significance, the social ability was better with lower income individual, while it was very less with no income individual (Table 6).

**Table 6:** Social Disability Based on Income SAQ - Satisfaction level questionnaire, MCQ - Masticatory ability questionnaire

Questionnaire	Income	Satisfied	Moderately Satisfied	Not Satisfied	Pearson Chi-Square P value
SAQ 3	Nil	0	1	0	0.062094
	3000	27	6	7	
	5000	19	7	1	
	8000	24	1	4	
	10000&above	13	6	5	
SAQ 7	Nil	0	1	0	0.112107
	3000	26	9	5	
	5000	19	3	5	
	8000	24	1	4	
	10000&above	13	6	5	
MCQ 10	Nil	0	1	0	0.535895
	3000	28	10	2	
	5000	19	8	0	
	8000	20	9	0	
	10000&above	18	6	0	
MCQ 11	Nil	0	1	0	0.316671
	3000	28	11	1	
	5000	20	7	0	
	8000	23	5	1	
	10000&above	22	2	0	
MCQ 13	Nil	0	1	0	0.220572
	3000	32	7	1	
	5000	20	6	1	
	8000	24	4	1	
	10000&above	14	10	0	

Functional limitation

On postoperative assessment based on education, the satisfactory level for functional improvement was higher for Higher secondary educated individuals followed by primary education and graduate individuals. The distribution of sample was Higher secondary-49, primary - 25 and graduate - 9 for SAQ1, Higher secondary-51, primary - 23 and graduate - 8 for SAQ2. Based on masticatory ability, the distribution of sample was Higher secondary-47, primary - 28 and graduate - 10 for MCQ1, Higher secondary-42, primary - 21 and graduate -8 for MCQ2, Higher secondary-46, primary - 26 and graduate - 7 for MCQ3, Higher secondary-40, primary - 27 and graduate - 6 for MCQ4, Higher secondary-52, primary - 25 and graduate - 10 for MCQ5, Higher secondary-57, primary - 23 and graduate - 13 for MCQ6 and Higher secondary-46, primary - 23 and graduate - 8 for MCQ7. Though there was no statistical significance, the functional improvement was better with Higher secondary education level (Table 7).

**Table 7:** Functional Limitation Based on Education SAQ - Satisfaction level questionnaire, MCQ - Masticatory ability questionnaire

Questionnaire	Education level	Satisfied	Moderately Satisfied	Not Satisfied	Pearson Chi-Square P value
SAQ1	Primary	19	7	7	0.898575
	Higher Secondary	40	18	16	
	Graduate	10	2	3	
SAQ2	Primary	22	7	4	0.819816
	Higher Secondary	42	17	15	
	Graduate	9	4	2	
MCQ1	Primary	28	5	2	0.082518
	Higher Secondary	47	27	2	
	Graduate	10	5	1	
MCQ2	Primary	21	11	1	0.874791
	Higher Secondary	42	30	2	
	Graduate	8	7	0	
MCQ3	Primary	26	5	2	0.136418
	Higher Secondary	46	26	2	
	Graduate	7	7	1	
MCQ4	Primary	27	6	0	0.032246
	Higher Secondary	40	31	3	
	Graduate	6	8	1	
MCQ5	Primary	25	8	0	0.712573
	Higher Secondary	52	20	2	
	Graduate	10	4	1	
MCQ6	Primary	23	9	1	0.019939
	Higher Secondary	55	17	2	
	Graduate	5	10	0	
MCQ7	Primary	23	10	0	0.612193
	Higher Secondary	46	26	2	
	Graduate	8	6	1	

On postoperative assessment based on employment status, the satisfactory level for functional improvement was higher for employed individuals followed by unemployed and pensioner individuals. The distribution of sample was Employed-49, Unemployed - 10 and Pensioner - 13 for SAQ1 and Employed-49, Unemployed - 12 and Pensioner - 14 for SAQ2. Based on masticatory ability, the distribution of sample was Employed-56, Unemployed - 19 and Pensioner -13 for MCQ1, Employed-50, Unemployed - 12 and Pensioner - 11 for MCQ2, Employed-57, Unemployed - 14 and Pensioner -12 for MCQ3, Employed-50, Unemployed - 15 and Pensioner -12 for MCQ4, Employed-60, Unemployed - 18 and Pensioner -13 for MCQ5, Employed-62, Unemployed - 16 and Pensioner -9 for MCQ6 and Employed-54, Unemployed - 17 and Pensioner - 11 for MCQ7. Though there was no statistical significance, the functional improvement was improved with employed individuals (Table 8).

**Table 8:** Functional Limitation Based on Employment SAQ - Satisfaction level questionnaire, MCQ - Masticatory ability questionnaire

Questionnaire	Employment Status	Satisfied	Moderately Satisfied	Not Satisfied	Pearson Chi-Square P value
SAQ1	Unemployed	10	9	6	0.16965
	Employed	49	14	20	
	Pensioner	13	4	2	
SAQ2	Unemployed	12	10	3	0.228617
	Employed	49	18	16	
	Pensioner	14	3	2	
MCQ1	Unemployed	19	6	6	0.717146
	Employed	56	27	2	
	Pensioner	13	6	2	
MCQ2	Unemployed	12	12	1	0.772695
	Employed	50	31	2	
	Pensioner	11	8	0	
MCQ3	Unemployed	14	11	0	0.456581
	Employed	57	22	4	
	Pensioner	12	6	1	
MCQ4	Unemployed	15	10	0	0.852958
	Employed	50	30	3	
	Pensioner	12	6	1	
MCQ5	Unemployed	18	7	0	0.851833
	Employed	60	21	2	
	Pensioner	13	5	1	
MCQ6	Unemployed	16	9	0	0.062266
	Employed	62	18	3	
	Pensioner	9	10	0	
MCQ7	Unemployed	17	8	0	0.676367
	Employed	54	26	3	
	Pensioner	11	8	0	

On postoperative assessment based on income, the satisfactory level for functional improvement were higher for low income individuals followed by upper middle class, lower middle class and higher class individuals. The distribution of sample was lower income class -30, upper middle class -21, lower middle class-14 and higher class - 8 for SAQ1 and lower class -35, upper middle class -13, lower middle class-10 and higher class - 9 for SAQ2. Based on masticatory ability, the distribution of sample was lower class -38, upper middle class -25, lower middle class-13 and higher class - 11 for MCQ1, lower class -36, upper middle class -28, lower middle class-15 and higher class - 11 for MCQ2, lower class -35, upper middle class -30, lower middle class-23 and higher class - 7 for MCQ3, lower class -42, upper middle class -30, lower middle class-21 and higher class - 10 for MCQ4, lower class -41, upper middle class -20, lower middle class-15 and higher class - 9 for MCQ5, lower class -33, upper middle class -20, lower middle class-15 and higher class - 9 for MCQ6 and lower class -35, upper middle class -17, lower middle class-15 and higher class - 11 for MCQ7. Though there was no statistical significance, the functional improvement was better with lower income individual, while it was very less with no income individual (Table 9).

**Table 9:** Functional Limitation Based on Income SAQ - Satisfaction level questionnaire, MCQ - Masticatory ability questionnaire

Questionnaire	Income	Satisfied	Moderately Satisfied	Not Satisfied	Pearson Chi-Square P value
SAQ 1	Nil	1	0	1	0.715441
	3000	30	15	12	
	5000	14	3	2	
	8000	21	10	2	
	10000&above	8	1	0	
SAQ 2	Nil	2	1	1	0.753563
	3000	35	27	18	
	5000	10	2	1	
	8000	13	1	0	
	10000&above	9	1	2	
MCQ 1	Nil	2	1	1	0.738232
	3000	38	18	10	
	5000	13	10	2	
	8000	25	11	1	
	10000&above	11	1	1	
MCQ 2	Nil	1	0	1	0.990977
	3000	36	23	3	
	5000	15	10	3	
	8000	28	20	1	
	10000&above	11	1	0	
MCQ 3	Nil	1	0	1	0.738682
	3000	35	9	1	
	5000	23	13	5	
	8000	30	13	1	
	10000&above	7	1	0	
MCQ 4	Nil	1	2	1	0.983131
	3000	42	18	0	
	5000	21	10	4	
	8000	30	10	1	
	10000&above	10	1	0	
MCQ 5	Nil	1	1	0	0.764083
	3000	41	10	0	
	5000	15	18	3	
	8000	20	4	1	
	10000&above	9	1	0	
MCQ 6	Nil	3	1	1	0.763359
	3000	33	9	0	
	5000	15	13	3	
	8000	20	11	0	
	10000&above	9	2	1	
MCQ 7	Nil	3	1	1	0.97878
	3000	35	4	0	
	5000	15	13	3	
	8000	17	4	0	
	10000&above	11	1	1	

## Discussion

Psychological assessments of patients have been found to be without influence on patients' judgment of dentures, whereas, others have been reported to distinguish significantly between satisfied and dissatisfied denture wearers [13,14]. Several studies demonstrated that the patient’s judgment can be predicted by information related to patient perceptions, expectations, and prior experiences [15]. Denture quality is defined in relation to a number of areas difficult to assess and no generally accepted standards exist. Accordingly, the validity and reliability of recordings of the quality of complete dentures are often doubtful [16]. Edentulism is considered a handicap with impacts on quality of life and nutrition. Provision of new complete dentures improves oral health-related quality of life. Patient’s satisfaction with their dentures is likely to be affected by their ability to perform certain tasks with them [17]. The present study was done to evaluate whether education level and socioeconomic status have an effect on the satisfaction level of the patient. Studies in edentulous subjects strongly support the concept that patient-based measures are more sensitive than functional measures for detecting differences between treatments [18].

The present study revealed that patient satisfaction was better with employed individuals but with the low-income group compared to the high-income group. In addition, the secondary level of educated individual had better satisfaction. This is contradictory to Poljak-Guberina et al. who found that age, education, marital status, income state, size of the residence and regional affiliation did not have a significant influence on satisfaction of patients with the prosthesis [19]. Also, not wearing prostheses was not linked to neuroticism. On the contrary, some researchers found no relationship between denture satisfaction and personality [20]. However, they used incomprehensive personality tests and paid little attention to reliability, validity, and suitability of the used tests. Moreover, Lowental and Tau found no relation between denture satisfaction and personality found no relationship between denture satisfaction and personality when denture satisfaction was assessed using denture satisfaction questionnaire [21].

## Conclusions

Rehabilitation of an elderly individual not only includes clinician skills but also the personal perception by the patient. The study concludes that though there was no statistically significant difference, the individual with a secondary level of education and with employed low socioeconomic status had a better denture satisfaction than the other categories.
